# Outlines of the Nerves, with Short Descriptions, Designed for the Use of Medical Students; and Outlines of the Arteries, with Short Descriptions, Designed for the Use of Medical Students

**Published:** 1852-04

**Authors:** 


*Outlines of the Nerves, with short descriptions, designed for the use of Medical Students ;* and *Outlines of the Arteries with short descriptions, designed for the use of Medical Students.* By JOHN NEILL, A. M., M. D., Surgeon to Wills Hospital, Demonstrator of Anatomy in the University of Pennsylvania. Second edition, Philadelphia, Barrington & Haswell, 1852.

We are glad to see that the favorable prognostications in relation to the early edition of these convenient "hand books" have been realized by a call for a new edition. As guides to the student in the lecture and dissecting room, we most cheerfully recommend them.

